# Replication and discovery of musculoskeletal QTLs in LG/J and SM/J advanced intercross lines

**DOI:** 10.14814/phy2.13561

**Published:** 2018-02-26

**Authors:** Ana I. Hernandez Cordero, Peter Carbonetto, Gioia Riboni Verri, Jennifer S. Gregory, David J. Vandenbergh, Joseph P. Gyekis, David A. Blizard, Arimantas Lionikas

**Affiliations:** ^1^ School of Medicine Medical Sciences and Nutrition University of Aberdeen Aberdeen United Kingdom; ^2^ Research Computing Center and Department of Human Genetics University of Chicago Chicago Illinois; ^3^ Department of Biobehavioral Health The Penn State Institute for the Neurosciences Molecular and Cellular Integrative Biosciences Program The Pennsylvania State University University Park Pennsylvania; ^4^ Department of Biobehavioral Health The Pennsylvania State University University Park Pennsylvania

**Keywords:** Bone, gene expression, QTL, Skeletal muscle

## Abstract

The genetics underlying variation in health‐related musculoskeletal phenotypes can be investigated in a mouse model. Quantitative trait loci (QTLs) affecting musculoskeletal traits in the LG/J and SM/J strain lineage remain to be refined and corroborated. The aim of this study was to map muscle and bone traits in males (*n* = 506) of the 50th filial generation of advanced intercross lines (LG/SM AIL) derived from the two strains. Genetic contribution to variation in all musculoskeletal traits was confirmed; the SNP heritability of muscle mass ranged between 0.46 and 0.56; and the SNP heritability of tibia length was 0.40. We used two analytical software, GEMMA and QTLRel, to map the underlying QTLs. GEMMA required substantially less computation and recovered all the QTLs identified by QTLRel. Seven significant QTLs were identified for muscle weight (Chr 1, 7, 11, 12, 13, 15, and 16), and two for tibia length, (Chr 1 and 13). Each QTL explained 4–5% of phenotypic variation. One muscle and both bone loci replicated previous findings; the remaining six were novel. Positional candidates for the replicated QTLs were prioritized based on in silico analyses and gene expression in muscle tissue. In summary, we replicated existing QTLs and identified novel QTLs affecting muscle weight, and replicated bone length QTLs in LG/SM AIL males. Heritability estimates substantially exceed the cumulative effect of the QTLs, hence a richer genetic architecture contributing to muscle and bone variability could be uncovered with a larger sample size.

## Introduction

Skeletal muscle is characterized by extensive individual variability; for instance, total muscle mass can range between 19 to 50 kg in healthy human males (Kim et al. 2002). Such variability may also impact metabolism and energy expenditure with possible implications for health (Elia 1992; Wolfe 2006). For example, low muscle mass is associated with reduced resting energy expenditure, which may contribute to obesity (Astrup et al. 1996). On the other hand, longevity of older adults is proportional to higher muscle mass (Srikanthan and Karlamangla 2014).

The skeleton provides structural support and serves as a mineral reservoir. Skeleton size is influenced by the growth hormone (GH) and insulin‐like growth factor 1 (IGF‐1) axis (Hindmarsh and Brook 1995; Rosenfeld 2005). The GH‐IGF‐1 axis affects growth of skeletal muscle in a similar way (Devol et al. 1990). However, growth of bone and muscle mass are not inseparable; for example, recent studies have demonstrated that linear growth of the bones is substantially influenced by the properties of chondrocytes (Fernandez‐Vojvodich et al. 2012), which can affect length but not girth of the muscle.

Individual variations in muscle mass and the size of the skeleton are attributed to environmental and genetic factors, as well as their interaction. Approximately half of the variation in muscle properties is due to poorly understood genetic factors (Silventoinen et al. 2008). Similarly, genetic variation plays a role in determining bone properties. For example, (narrow‐sense) heritability of tibia and femur size has been estimated to be 73% and 66%, respectively (Chinappen‐Horsley et al. 2008).

The genetics underlying variation in health‐related musculoskeletal phenotypes can be investigated in mouse models (Lionikas et al. 2010; Carbonetto et al. 2014; Parker et al. 2016). The LG/J and SM/J strains were independently derived by selection for large (Goodale 1938; Tassano et al. 2013) and small body size (MacArthur 1944; Chai 1956), respectively, and offer a particularly useful model. Specifically, these strains differ in long‐bone length, biomechanical and structural proprieties of the bones (Reich et al. 2008; Norgard et al. 2011), metabolic traits (Nikolskiy et al. 2015), muscle mass (Lionikas et al. 2010, 2012), and characteristics of muscle fibers (Carroll et al. 2012, 2017). Advanced intercross lines (AIL) of these two strains (LG/SM) have been developed to facilitate more effective investigation of the genetic factors underlying phenotypic differences (Reich et al. 2008; Lionikas et al. 2010; Rai et al. 2015). An AIL approach was first proposed by Darvasi and Soller (1995) in order to improve mapping resolution of genome‐wide association studies (GWAS). Each consecutive generation of AIL leads to additional recombination and improves mapping resolution of quantitative trait loci (QTLs) (Flint and Mott 2001). To corroborate and refine the QTLs affecting muscle mass and bone morphology reported in the LG/SM lineage (Lionikas et al. 2010; Carbonetto et al. 2014), we studied an LG/SM AIL that has been maintained for over 50 generations. The genomes of the LG/J and SM/J strains have been sequenced (Nikolskiy et al. 2015) and the transcriptional profiles of skeletal muscle are available (Lionikas et al. 2012), which facilitates identification of genes underlying the QTL.

We used the software GEMMA (Zhou and Stephens 2012) to fit a linear mixed model (LMM) and evaluate evidence for association at 7236 SNPs on autosomal chromosomes as well as the X chromosome. GEMMA and other LMM‐based QTL mapping approaches have emerged as robust strategy for QTL mapping in mouse genetics studies because LMMs are able to reduce inflation of association test statistics due to close relationships or population structure (Joo et al. 2016; Parker et al. 2016). However, GEMMA does not model dominance effects, and we sought to understand whether this would meaningfully reduce power to detect QTLs on autosomal chromosomes.

We report here the replication and refinement of muscle and bone QTLs identified in the 50th‐generation LG/SM AIL. We prioritized positional candidate genes base on two criteria: (1) presence of nonsynonymous polymorphisms within the coding sequence of the genes and (2) presence of polymorphisms in regulatory elements (promoter and enhancers) located within the vicinity of the positional candidate gene. We also analyzed gene expression in the LG/SM AIL samples to further prioritize candidates for the skeletal muscle weight locus 34 (*Skmw34)* which was identified in the previous study (Lionikas et al. 2010).

## Materials and Methods

All procedures and experiments on the mice used for this research were executed according to the Institutional Animal Care and Use Committee of the Pennsylvania State University (PSU).

### Animals

All mice in the AIL derive from the LG/J and SM/J strains (“LG/SM AIL”), and were initiated by Dr. Cheverud at the Washington University, St. Louis (Cheverud et al. 1996). Dr. Palmer imported animals from all available litters of an F33 generation to establish a colony at the University of Chicago. For this study, males (*n* = 73) were imported to PSU from Dr. Palmer and females (*n* = 63) were imported from Dr. Cheverud at the F49 generation. These animals were crossed to produce the study population. The largest and smallest males in each litter (excluding runts) were used in the study of male mice (*n* = 506). The study population was produced over five generations, F50‐F54. The males from Washington University were mated with the females from Chicago to produce the first generation of test animals. Surplus animals from these litters were used to form matings for the next generation. A pseudo random procedure was followed avoiding matings between animals with a grandparent in common.

Relationships among individuals were estimated using the genetic markers (Carbonetto et al. 2014); genetic markers can yield more precise estimates of genome sharing than the pedigree (Parker et al. 2014; Speed and Balding 2015). See below for more details on calculation of marker‐based relatedness estimates.

All animals were housed at room temperature (70–72°F) at 12:12 h light–dark cycle, with 1–4 same‐sex animals per cage and with ad libitum access to chow (Purina LabDiet 5001) and water.

A total of 14 mice were not included due to genotyping or phenotyping errors, resulting in a total of 492 LGSM AIL samples being used in the final analysis.

### Phenotypes

All mice were killed at ~12 weeks of age and one hindlimb was removed. Four muscles and one bone were dissected from the hindlimb: tibialis anterior (TA), extensor digitorum longus (EDL), gastrocnemius (gastroc), soleus, and the tibia bone. The procedure was executed under a dissection microscope. The muscles were weighed to 0.1‐mg precision on a balance (Pioneer, Ohaus). Tibia length was measured (mm) using an electronic digital calliper (Powerfix, Profi).

Since dissections of the muscles were performed in two facilities, systematic differences were expected, and were accounted for in our analyses (see Covariates).

### Genotypes

All mice were genotyped using the MEGA Mouse Universal Genotyping Array (MegaMUGA) for 75,746 SNPs (73,301 on the autosomes and 2386 on X and Y). Hardy–Weinberg equilibrium (HWE) tests were conducted to ensure the quality of the genotypes. Markers that were not in HWE (*P* = 0.01) were checked based on their allele frequency and call rate, and we retained those that had acceptable minor allele frequencies (MAF > 0.20) and a call rate >95%. A subset of 7236 SNPs (7187 SNPs on autosomes and 49 SNPs on the X chromosome) polymorphic between the two strains (LG/J and SM/J) was retained for the genome‐wide association analyses. The median distance between adjacent SNPs used was 126.9 Kb, and the maximum inter‐SNP interval was 15 Mb, except for chromosome (Chr) 8, 10, 14, and X, which had a maximum inter‐SNP interval of 19, 16, 16, and 33 Mb, respectively. 3320 SNPs that appeared as if the alleles were taken from the complementary strand were retained and the genotypes were swapped. The mean correlation among adjacent genotypes was 0.93.

Genetic distances were estimated in cM using Jackson Laboratories' Mouse Map Converter tool (http://cgd.jax.org/mousemapconverter/) (Cox et al. 2009), this was required to estimate missing genotypes for analyses executed with QTLRel (Cheng et al. 2011).

### Mapping

The different degrees of relatedness among mice, often called “cryptic relatedness,” can inflate phenotype–genotype association test statistics in QTL mapping, leading to undesirably high rate of spurious associations. To control for inflation due to cryptic relatedness, an approach based on linear mixed models (LMMs) was used. This approach has emerged as a robust strategy to account for confounding effects occurring due to population structure and cryptic relatedness (Yu et al. 2006; Cheng et al. 2010; Yang et al. 2014).

We compared two LMM methods for QTL mapping: GEMMA (Zhou and Stephens 2012) and QTLRel (Cheng et al. 2011). In QTLRel, the LMM is defined as follows:

$$y_{i}=\mu +c_{i}\beta_{c}+a_{\mathrm{ij}}\beta_{j}+d_{\mathrm{ij}}\beta_{\mathrm{dj}}+p_{i}+\varepsilon_{i}$$

where *y* is the phenotype for individual *i*,* μ* is the sample mean and *c*_*i*_ represents the vector of covariates (defined below). The QTLRel model includes an additive genotype term, *a*_*ij*_ (*n* vector of markers genotypes, where *n* is the number of samples and genotypes were encoded as 0, 1, or 2 for homozygous LG, heterozygous, and homozygous SM allele, respectively), and a dominance term (*d*_*ij*_) for each SNP *j*. The additive and dominance effects for each SNP were denoted by *β*_j_ and *β*_dj_, respectively. The polygenic term *p*_i_ is meant to capture variation in the phenotype explained by overall differences in genetic relatedness; *p*_i_ is taken be a random vector drawn from a multivariate normal distribution with n *x* n covariance matrix *K*, where *n* is the number of samples. The general expression for the covariance matrix of the polygenic effect is given by Cheng et al. (2010). The *K* matrix was estimated from the genotype data (more details are given below). The last term in the model captures any additional phenotypic variance (e.g., due to environmental factors) that is not explained by the covariates and genetic variation.

The GEMMA software defines the LMM in the same way, except that it does not include a dominance effect. GEMMA is able to fit an LMM with faster execution of the computational analyses (Zhou and Stephens 2012). Note that this formulation does not account for nonadditive genetic effects. However, in practice the nonadditive variance components typically make negligible contributions to the total phenotype variation in an outbred population, hence would be expected to have little impact on the final results of the QTL mapping (Abney et al. 2000; Carbonetto et al. 2014). All analyses were executed using the same computer with 8 GB of memory and a 2.9 GHz Intel Core i7 processor.

Note that a dominance effect is not needed to map QTLs on the X chromosome in males (hemizygote), therefore we only used GEMMA to map QTLs on the X chromosome.

### Covariates

The geographical site of tissue harvesting/collection (Penn State or Aberdeen), which also encompasses the two trained individuals that collected the tissues in each facility, had a statistically significant effect on the muscle weights (*P* < 0.05) and the tibia length (*P* = 4.69e^−11^), and was used as a covariate to adjust for systematic differences. Similarly, tibia length was significantly (*P* < 2.2e^−16^) and positively correlated with the muscle weights. Tibia length explained 39%, 38%, 41%, and 22% of the phenotypic variability observed on TA, EDL, gastrocnemius, and soleus muscles, respectively. By controlling for the effect of tibia length, the aim was to capture genetic effects on variation in muscle tissue that could not be explained by variation in bone size, that is, muscle tissue‐specific effects (Parker et al. 2016). Therefore, we included the site of tissue harvesting/collection and the tibia length as covariates for the GWAS analyses on the four muscles. For the analyses of the tibia length, the site of tissue harvesting/collection was used as a covariate. Age was not included as a covariate as all mice were of similar age (~12 weeks).

### Genetic relatedness

Genotype data were used to define the n *x* n covariance matrix *K* for the polygenic term in the LMM. This matrix was constructed as described by Parker et al. (2016) and was defined as *K* = *XX*'/*P*, where *P* is the number of markers and *X* is the genotype matrix; the LG homozygote, heterozygous, and SM homozygous alleles were encoded in the genotype matrix as 0, 1, and 2, respectively. A useful property of this formulation of the “realized relationship” matrix in an AIL is that it has a close interpretation to kinship coefficients.

A critical consideration for LMM‐based association mapping is that including a genetic marker in the relatedness matrix *K* can deflate the test statistic for this marker, leading to a loss of power to detect a QTL. This phenomenon has been called “proximal contamination”, and depends on the generation of the individual in an AIL (Listgarten et al. 2012). Therefore, each time we analyzed a chromosome we excluded its markers from the relatedness matrix *K* to avoid proximal contamination; this “leave one chromosome out” approach is widely used (e.g., Lippert et al. 2011; Listgarten et al. 2012; Yang et al. 2014).

### SNP heritability

We used GEMMA to estimate the proportion of phenotypic variance explained by all available genotypes, often called “SNP heritability,” using the same *n x* n realized relatedness matrix *K* defined above. As part of the QTL mapping, GEMMA fits a “null” model that also provides an estimated for SNP heritability. In the Results section we report these estimates along with their estimated standard error.

### LOD scores and significance thresholds

The LOD score or, equivalently, the base‐10 logarithm of the likelihood ratio, was estimated for each SNP. Although GEMMA does not provide LOD scores directly, we were able to derive LOD scores from the *P*‐values computed by GEMMA. We reported support for association using LOD scores instead of *P*‐values in order to more easily compare against previous QTL mapping results from the same lineages (Lionikas et al. 2010).

We calculated a threshold for significance by estimating the distribution of maximum LOD scores under the null hypothesis, then we took the threshold to be the 100(1‐α) the percentile of this distribution, with *α* = 0.05 as a “significant” threshold (Abiola et al. 2003). A common approach to estimating the null distribution is to randomly permute the phenotype observations while keeping the genotypes the same. Such a procedure is technically not appropriate here because it fails to account for the lack of exchangeability among the samples due to varying relatedness. However, Carbonetto and colleagues (Carbonetto et al. 2014) estimated thresholds in an AIL with and without taking relatedness into consideration and found at most a small differences in 0.15 LOD between them. We tested the effect of the population structure on one of the traits, TA muscle, by estimating the threshold with and without the relatedness matrix. The outcome of this analysis showed a 0.08 LOD difference between the two estimates, indicating that accounting for cryptic relatedness does not appear to have a major impact on assessment of significance thresholds in AILs. Hence we used less computationally demanding, naive permutation test. To estimate the null distribution we randomly permuted the phenotypes while keeping the genotypes the same. We used 1000 permutation replicates to estimate the distribution following Churchill and Doerge (1994). Importantly though, we included the population structure in the LMM used to test the associations in order to limit inflation of false positives.

The same approach described above was used to calculate the X chromosome threshold of significance, although, a different number of permutations were used to estimate the distribution of maximum LOD scores. Based on the scheme described by Broman et al. (2006) the adequate number of permutations for X chromosome was estimated as *L*/{*L*_*X*_} times more permutation replicates to get the same precision as used for the rest of the genome, where $L=\sum_{i}L_{i}$ is the sum of the length of each chromosome *i* and *L*_X_ is the length of the X chromosome.

### QTL support intervals

Genes close to the QTL's peak SNP are often good candidates to explain the phenotypic differences in the investigated trait. We used a 1.5‐LOD support interval to approximate the confidence interval for the location of the QTL. The primary reason we chose this interval was to provide a reasonable interval that is consistent with previous studies using AIL mice (Lionikas et al. 2010; Parker et al. 2014).

### Software and data availability

The full code and data reproducing the steps of the analyses are available for download at https://github.com/AnaIHer/LGSM-AIL.

### Bioinformatics databases used to evaluate candidate genes

To identify promising candidate genes, we examined all genes within the 1.5‐LOD support interval of each significant QTL. First, we compared the expression of these genes in skeletal muscle and bone cells to other tissues and cell types (BioGPS database, http://biogps.org; Gene Expression Omnibus, https://www.ncbi.nlm.nih.gov/geo/), with a focus on genes that are abundantly expressed in the tissues of interest (muscle and/or bone). Second, we also examined differences in mRNA abundance between the LG/J and SM/J strains in muscle tissue (Lionikas et al. 2012). Third, we evaluated possible effects of nonsynonymous SNP and homozygous indel variants between the LG/J and SM/J strains on protein function and regulatory elements (Nikolskiy et al. 2015). Genes within QTL support interval meeting at least one of the selection criteria were consider positional candidate genes. Note that skeletal muscle gene expression was used as a selection criteria only for the muscle candidate genes.

### Quantitative RT‐PCR

The AIL animals, 22 in total, homozygous for the LG/J or SM/J allele in the region of the candidate genes were selected for gene expression analyses. RNA isolation from TA muscle, snap‐frozen immediately after dissection in liquid nitrogen chilled isopentane, was carried out using TRIzol (Invitrogen Life Technologies, Carlsbad). Quantification of total RNA was performed on a NanoDrop 1000 spectrophotometer (Thermo Scientific) and quality tested on an Agilent Tapestation with R6K Screentapes. The RNA integrity number ranged between 5.5 and 9.4, with a median of 8.8. Two micrograms of RNA was used for cDNA synthesis by SuperScript III reverse transcriptase (Invitrogen, Carlsbad). Primers for *Mical2* (forward: GCAAACAGCGAAAGAGACG; reverse: CCTGCTCCTGCCATGTTC), *2310014F06Rik* (forward: AAAGCACTTGCTGCTCTGC; reverse: GGATGGATGCTAGAAGATTGGA), *Arntl* (forward: GCCCCACCGACCTACTCT; reverse: CTTTGTCTGTGTCCATACTTTCTTG), and *Sox6* (forward: GGAATTTGGACCCCTCTGA; reverse: AGCTGAGCGGCATAGAGC) genes were designed using the Universal ProbeLibrary Assay Design Center (www.roche-applied-science.com) to be compatible with FAM‐labeled probes #101, #92, #104 and #77 (Roche), respectively. The final reaction mix consisted of 2X LightCycler 480 Probes Master (Roche, Germany), 500 nmol/L of forward and reverse primers, 100 nmol/L of the FAM‐labeled probe (Roche) and contained 30 ng of cDNA. Mouse *GAPD* (20X) reagents (Applied Biosystems, UK) were used for the analyses of a reference gene. A quantitative PCR (qPCR) was carried out in replicates of 3 for each sample‐by‐gene combination using Roche LightCycler 480 (Roche Diagnostics LTD, Burgess Hill, UK). The efficiency of the qPCR was within the recommended range (Schmittgen and Livak 2008) and comparable among the four genes (between 1.78 and 1.99). Quantification of gene expression was performed using the comparative Ct method (Pfaffl 2001). Gene expression of homozygous mice (LG/LG vs. SM/SM) was assessed using the Mann–Whitney test.

## Results

### Phenotype distribution

**Figure 1:**
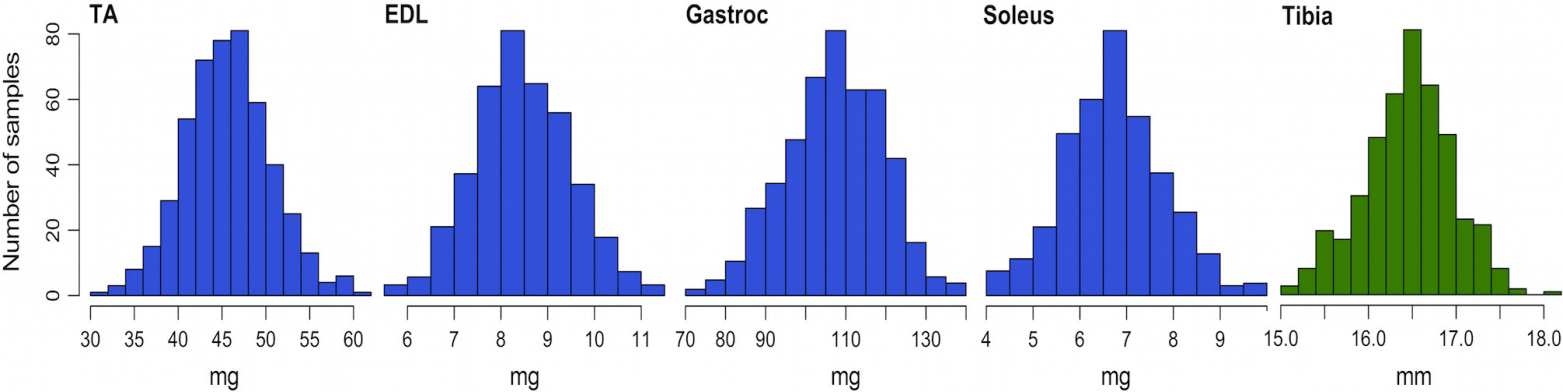
Empirical distribution of traits investigated in LG/SM AIL male mice. Blue histograms represent muscle traits. Tibialis anterior (TA), extensor digitorum longus (EDL), gastrocnemius (Gastroc) and soleus weights differ greater than twofold between the most and least muscular animals. Green histogram represent tibia.

The muscle weight data (TA, EDL, Gastroc, and Soleus) and bone trait data (tibia length) from the LG/SM AIL are summarized in Table 1. The traits were highly variable, exhibiting over twofold difference in muscle weight, and substantial variability in tibia length (Fig. 1).

**Table 1:** Proportion of variance in muscle and bone traits explained by measured genetic variants in LG/SM AIL male mice

Traits	SNP heritability ± SE	Mean ± SD (*n* = 492)
TA, mg	0.56 ± 0.06	45.7 ± 4.9
EDL, mg	0.51 ± 0.06	8.5 ± 1.0
Gastroc, mg	0.45 ± 0.06	107.1 ± 12.0
Soleus, mg	0.46 ± 0.06	6.7 ± 1.0
Tibia length, mm	0.40 ± 0.06	16.4 ± 0.5

Columns form left to right: (1) trait investigated and units; (2) proportion of phenotypic variance explained by the 7,236 available genetic markers on autosomal chromosomes and X chromosome (“SNP heritability”) and standard error for the SNP heritability estimates. (3) Sample mean and standard deviation of the phenotype.

All muscle weights were highly correlated with each other (Table 2). TA and EDL muscles showed the strongest correlation (*r* = 0.8). The soleus exhibited the weakest correlation with other muscles (*r* = 0.6–0.7). These correlations are consistent with correlations measured in other mouse populations (Lionikas et al. 2010). The tibia showed a moderate correlation with all muscles (*r* = 0.4–0.6).

**Table 2:** Sample correlations (*r*) among all traits measured in the LG/SM AIL

Trait	TA	EDL	Gastroc	Soleus	Tibia
TA	1.00	0.80	0.79	0.60	0.65
EDL		1.00	0.81	0.61	0.64
Gastroc			1.00	0.67	0.66
Soleus				1.00	0.49
Tibia					1.00

TA, tibialis anterior; EDL, extensor digitorum longus; Gastroc, gastrocnemius.

### QTLs affecting muscle and bone

Before mapping QTLs for these traits, we first assessed the overall genetic contribution to variation in muscle and bone traits. The “SNP heritability” estimates of muscle mass (*i.e*., proportion of variance in muscle mass explained by the available SNPs) ranged between 0.46 and 0.56; tibia length was 0.40 (Table 1).

**Figure 2:**
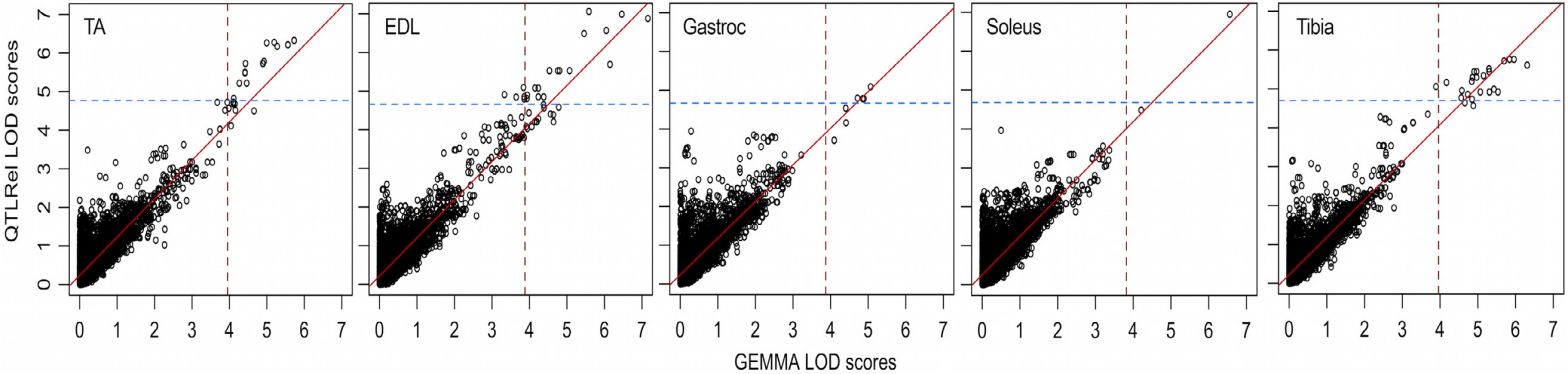
Comparison of LOD scores at all 7236 candidate SNPs computed using QTLRel and GEMMA. Horizontal and vertical dashed lines depict significance thresholds (*α* = 0.05) for QTLRel and GEMMA LOD scores, respectively.

We mapped QTLs affecting variability in muscle and bone traits. We computed LOD scores at all SNPs using two linear‐mixed model approaches, QTLRel (Cheng et al. 2011) and GEMMA (Zhou and Stephens 2012). (Note we only applied GEMMA to SNPs on the X chromosome; see Methods). A key difference is that QTLRel estimates a dominance effect for each SNP, whereas GEMMA only estimates a single (additive) effect at each SNP. Consistent with previous reports (Zhou and Stephens 2012), the QTL mapping analysis using GEMMA was approximately 10 times faster than the QTLRel‐based analysis 161 sec versus 1628 sec, respectively, when running the programs on the same 2.9 GHz Intel Core i7 processor. The overall support for QTLs was broadly similar for all phenotypes using both methods (Fig. 2). QTLRel yielded somewhat higher LOD scores on average because the additional model parameter allows QTLRel to obtain a better fit to the data. This outcome suggests that including dominance effects in the LMM results in limited gain of QTL detection power in an AIL.

**Figure 3:**
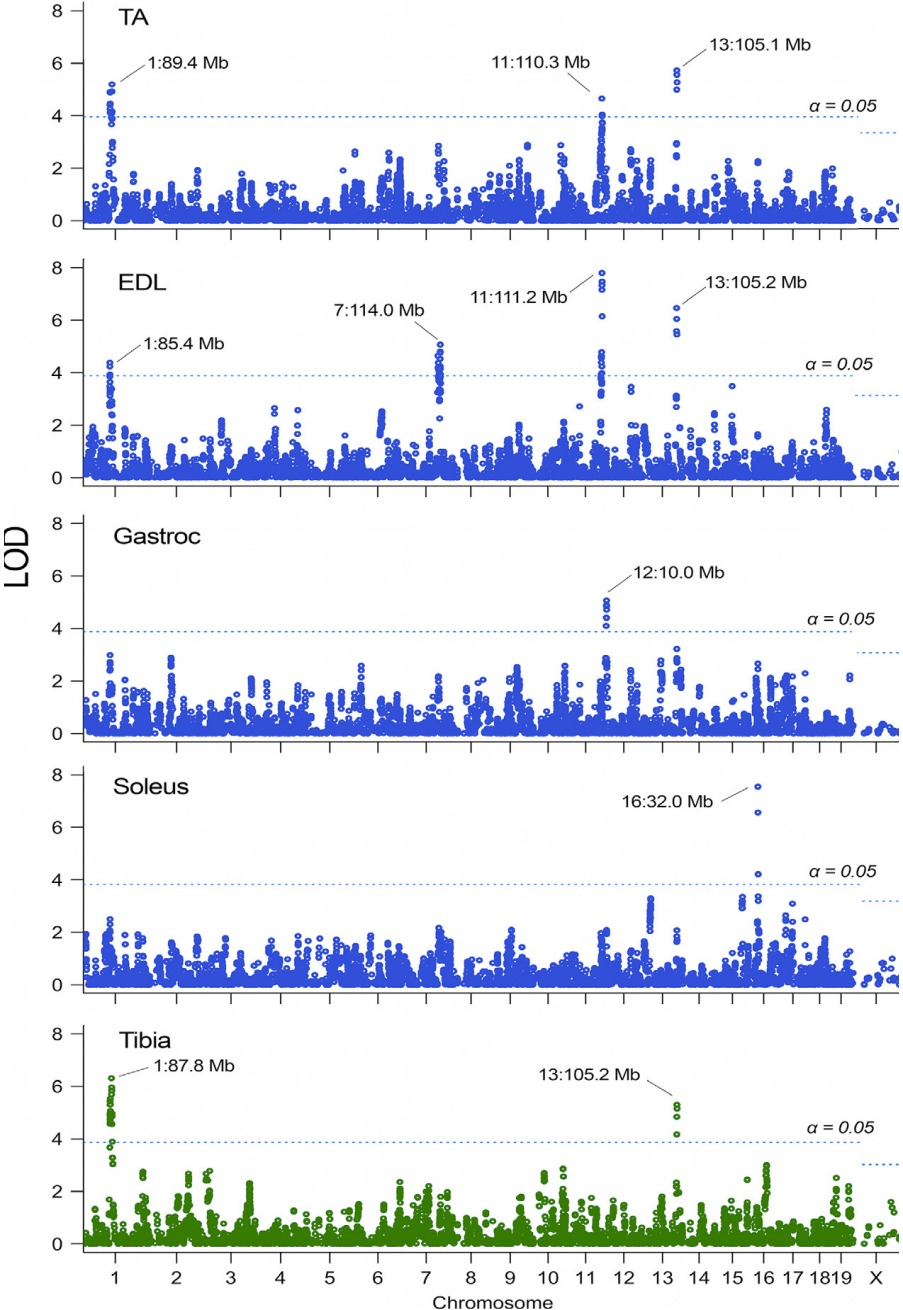
Genome‐wide scans for TA, EDL, gastrocnemius and soleus muscle weight (blue), and Tibia length (green). The vertical axes show the GEMMA LOD scores for the 7236 polymorphic SNPs arranged by their chromosomal positions in the horizontal axis. The horizontal blue line represents threshold at *α* = 5%.

Among the four muscle traits, 6 QTLs contributing to muscle weight variation exceeded the permutation‐derived significance threshold (LOD thresholds for TA, EDL, Gastroc, and soleus were 3.95, 3.88, 3.87, and 3.82, respectively) on Chr 1, 7, 11, 12, 13, and 16 (Fig. 3). The median, maximum, and minimum 1.5‐LOD QTL support intervals were 2.8 Mb, 9.0 Mb, and 0.8 Mb, respectively (Table 3). No QTLs were found on the X chromosome in any of the investigated traits. The QTL on Chr 7 was denoted as a “replicated” locus since this locus is located within previous QTL support interval (Lionikas et al. 2010). The remaining QTLs found here were identified for the first time and we referred to them as “novel”.

**Table 3:** Quantitative trait loci affecting muscle weights and tibia length in LG/SM AIL male mice

QTL	Chr	QTL region, Mb	Top SNP, Mb	Trait	Genes	Size, Mb	LOD	PVE (%)	Mean trait (mg) LG/Het/SM	*β*_1_	*β*_2_	*β*_3_	Ref
*Skmw43*	1	85.4–88.6	89.4	TA	132	3.1	5.19	4.0	45.5/45.9/46.2	−1.34	−1.39	0.13	
	1	85.4–87.9	85.4	EDL		2.5	4.38	3.2	8.5/8.6/8.5	−0.27	−0.22	0.07	
***Lbn1.1b***	1	85.4–87.9	87.8	Tibia	100	2.5	6.30	3.3	16.5/16.4/16.2	0.19	0.16	−0.02	(Norgard et al. 2011)
***Skmw34***	7	106.0–115.0	114.0	EDL	241	9.0	5.07	5.5	8.6/8.6/8.2	0.23	0.24	0.08	(Lionikas et al. 2010)
*Skmw44*	11	109.4–112.1	110.3	TA	31	2.7	4.65	2.3	45.1/46.2/46.9	−1.25	−0.89	0.60	
	11	111.0–111.8	111.2	EDL		0.8	7.79	6.2	8.3/8.7/8.9	−0.34	−0.31	−0.01	
*Skmw45*	12	7.7–10.4	10.0	Gastroc	45	2.7	5.06	3.4	110.0/107.1/105.1	2.77	2.82	−0.61	
*Skmw46*	13	103.0–108.0	105.0	TA	40	5.0	5.72	3.8	45.7/45.6/46.2	−1.26	−1.48	−0.41	
	13	103.0–108.0	105.2	EDL		5.0	6.46	5.5	8.5/8.5/8.7	−0.29	−0.32	−0.09	
***Lbn13.1***	13	103.0–108.0	105.2	Tibia	40	5.0	5.29	6.3	16.6/16.4/16.2	0.16	0.15	−0.03	(Norgard et al. 2011)
*Skmw47*	16	30.9–32.9	32.0	Soleus	52	2.0	7.54	5.1	7.0/6.8/6.4	0.32	0.33	0.07	

Columns form left to right: (1) QTL name, bold names are replicated QTLs; (2) QTL chromosome, (3) QTL region based on 1.5 LOD support interval (build 38); (4) SNP position within QTL region with largest LOD score; (5) Trait investigated; (6) Number of genes within each QTL; (7) QTL width based on the 1.5‐LOD support interval; (8) LOD score for the SNP with the largest score; (9) Proportion of phenotypic variance explained (PVE) by the top SNP within the 1.5 LOD support interval after removing linear effects of the covariates. (10) Average phenotypic value for the alleles LG homozygous, heterozygous, and SM homozygous. (11) GEMMA beta estimate. (12) QTLrel beta estimate for the additive component. (13) QTLrel beta estimate for the dominance component. MAF for the QTLs peak markers was >0.22. (14) Reference for QTLs replicated in past studies.

Among the six QTLs affecting muscle weight, an increasing effect on the phenotypic mean was conferred by the LG/J allele (Chr 7, 12, and 16) or the SM/J allele (Chr 1, 11, and 13), although heterosis mode was apparent for EDL on Chr 1 (Table 3). We also searched for QTLs affecting muscle mass unadjusted for tibia length, but identified only one significant QTL (Chr 15:51.62 Mb) explaining 4.4% of phenotypic variance of EDL muscle. The LG allele conferred an increase in muscle weight in this case.

Two QTLs affecting tibia length were identified (LOD threshold > 3.96) in the LG/SM AIL males on Chr 1 and 13 (Fig. 3). Both QTL were treated as “replicated” since they are located within a previous QTL support intervals (Nicod et al. 2016). The QTL on Chr 13 overlapped with muscle weight QTL in the same genomic location (~105 Mb). An increasing effect on tibia length was conferred by the LG/J allele for both QTLs (Table 3). The genetic architecture of muscle weights and tibia length based on the analysis of the LG/SM AIL is summarized in Table 3.

### Candidate genes

For each QTL (Table 3), we screened all genes within the 1.5‐LOD interval to identify promising muscle and bone candidate genes. We used the following criteria for screening: (1) the gene was preferentially expressed in skeletal muscle and/or the C2C12 myogenic cell line, or bone (osteoclast, osteoblasts, and/or chondrocytes (Chau et al. 2014; Fukada et al. 2008; James et al. 2005, 2007)) in comparison to the other tissues/cell types; (2) nonsynonymous SNPs with potentially damaging effects on protein function (determined by in silico analysis (Nikolskiy et al. 2015)) were present; and (3) there were differences in gene expression in muscle tissue between the LG/J and SM/J strains (skeletal muscle gene expression was used as a selection criterion only for the muscle candidate genes). These screening criteria produced four candidate genes within the muscle weight QTL on Chr 7 (*Skmw34*), and four genes within the two tibia length QTLs (Table 4). Note that we identified potential candidate genes within all but one (*Skmw44*) QTL, however, we only focused on genes within the most robust, replicated loci.

**Table 4:** Candidate genes identified within muscle and bone QTLs in LG/SM AIL

Trait	Gene description	Gene symbol	Gene position, bp	Nonsyn SNP	Differential expression	Locus ID
Tibia	DIS3 like 3′‐5′ exoribonuclease 2	*Dis3l2*	chr1: 86703808‐87050095	X		Lbn1.1b
Tibia	Holliday junction recognition protein	*Hjurp*	chr1: 88262471‐88277633	X		*Lbn1.1b*
EDL	Microtubule associated monooxygenase calponin and LIM domain containing 2	*Mical2*	chr7:112225856‐112355194	X	LG/J>SM/J	*Skmw34*
EDL	RIKEN cDNA 2310014F06 gene	*2310014F06Rik*	chr7:112612560‐112680081		SM/J>LG/J	*Skmw34*
EDL	aryl hydrocarbon receptor nuclear translocator‐like	*Arntl*	chr7:113207465‐113314122		LG/J>SM/J	*Skmw34*
EDL	SRY (sex determining region Y)‐box 6	*Sox6*	chr7:115470872‐116038796	x		*Skmw34*
Tibia	Splicing regulatory glutamine/lysine‐rich protein 1interacting protein 1	*Srek1ip1*	chr13:104792484‐104839274	x		*Lbn.13.1*
Tibia	Small integral membrane protein 15	*Smim15*	chr13:108046424‐108049146	x		*Lbn13.1*

Genes listed in this table are located within replicated QTLs. Candidate genes were included in this table based on the presence of a nonsynonymous SNP in their coding sequence with a predicted effect on protein function (based on Poly‐phen2 or SIFT algorithm), differential expression in TA muscle between LG/J and SM/J mice strains (*P* < 0.10) or their known function.

**Figure 4:**
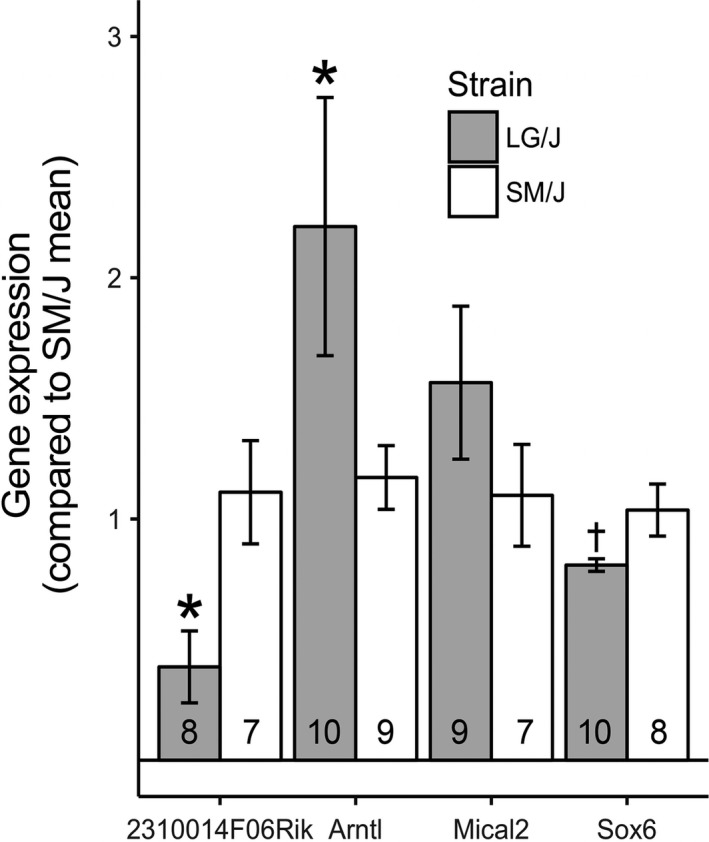
Allele‐dependent expression of candidate genes in TA muscle of the LG/SM AIL males. Homozygous carriers of the SM/J and LG/J alleles were compared using Mann–Whitney tests. Mean and SEM. *P < 0.05 (one‐tailed), †*P* < 0.05 (two‐tailed) compared to SM/J expression. Number indicates sample size within the genotypic group.

Subsequent analyses were performed to further prioritize among the four *Skmw34* locus genes (Table 4). We tested the hypothesis that an allelic variant of a gene is associated with its transcript abundance in TA muscle of the LG/SM AIL males. One‐tailed *P* values were used to evaluate allelic effect on expression of genes for which the direction of the expression difference was known (Lionikas et al. 2012); otherwise, two‐tailed *P* values were used (for *Sox6*). The qPCR analysis revealed that expression of *Sox6* and *2310014F06Rik* were significantly reduced (*P* < 0.03), whereas that of *Arntl* increased by ~twofold (*P* < 0.03) in the presence of LG/J compared to SM/J allele. Note we did not identify a statistically significant allelic effect on expression of *Mical2* gene (Fig. 4).

## Discussion

In this study, we mapped the genetic variants contributing to variation in muscle mass, and bone in LG/SM AIL male mice. A total of seven QTLs were mapped to regions with a median width of 3.1 Mb; three of these (Chr 1, 7, and 13) overlapped previously identified loci for muscle mass and tibia length (Lionikas et al. 2010; Norgard et al. 2011). Promising candidate genes were identified within the support interval of each replicated QTL. Within the *Skmw34* locus, expression analyses highlighted the *Arntl* and *2310013F06Rik* genes as the most plausible genes linked to muscle mass.

Male mice of the LG/SM AIL exhibited a large variability in muscle and bone traits (Fig. 1). Much of this variability is estimated to be explained by genetic factors; the SNP heritability ranged from 0.46 to 0.56 for muscle weights, and bone length SNP heritability estimate was 0.40. Due to targeted selection of the largest and smallest littermates for the study population (see Methods) these estimates might represent overestimates, albeit all of them are within range of reported SNP heritability values for corresponding traits in the LG/SM intercross (Lionikas et al. 2010; Norgard et al. 2011). Higher broad‐sense heritability estimates for muscle weight, ranging from 0.82 to 0.88, were reported in F2 and F34 LG/SM AIL mice (Lionikas et al. 2010). This discrepancy in heritability estimates may be attributed to two factors: (1) SNP heritability fails to account for the epistatic effects that may be contributing to the phenotypic variance (Zuk et al. 2012), or “polar overdominance” pattern of inheritance that can also affect skeletal muscle traits as demonstrated in sheep (Cockett et al. 1996); and (2) SNPs used in this study do not capture all causal variation in these traits (Yang et al. 2010; Wray et al. 2013).

We used two software packages, GEMMA and QTLRel, to map QTLs for muscle and bone weight QTLs. Comparison of the two software packages revealed that they yield similar QTL mapping outcomes (Table 3). This result suggests that fitting additive and dominance effects together in the LMM leads to only a modest gain of QTL detection power (Fig. 2). Therefore, a 10‐time higher computational speed afforded by GEMMA, which estimates additive effects only (Zhou and Stephens 2012), might be preferred for QTL mapping, particularly in larger populations.

Each of the six identified muscle QTLs individually explained 2–5% of variability in muscle weight (Table 3). Hence, the proportion of genetic variance accounted for by an individual QTL is between 5% and 10%. The aggregate effect of the 3 and 4 QTLs for TA (in total, 10% of phenotypic variance explained) and EDL (18%), respectively, amounted to one‐fifth and one‐third of the SNP heritability estimates for these muscles. Two tibia length QTLs were similar to the muscle QTLs with respect to the effect size and in aggregate (9%) accounted for nearly a quarter of the SNP heritability. Hence, a substantial fraction of the SNP heritability of all measured variables remains unaccounted for. Small size effects and a limited sample size may be contributing to the “missing heritability” in these complex traits (Manolio et al. 2009). Population size would have to be doubled to increase detection power to ~90% (Gatti et al. 2014).

The SM/J allele conferred an increasing effect compared to the LG/J allele in three out of six muscle QTLs (Chr 1, 11 and 13). Thus, although muscle weight of the LG/J mice is twofold greater compared to SM/J (Lionikas et al. 2010), the SM/J strain also carries increasing alleles. This is possible because these two strains were derived from the different founder populations (Goodale 1938; MacArthur 1944). Therefore accumulation of the increasing or decreasing variants over the course of selective breeding was determined by allelic diversity of the founders.

The QTL on Chr 7 overlapped the position of *Skmw34* locus identified in a previous mapping study (Lionikas et al. 2010). We also replicated two QTLs (Chr 1, 13) affecting tibia length in the same lineage (Norgard et al. 2011). The modest replication of muscle QTL is likely due to different covariates used in each study. In the present analysis, tibia length adjustment was applied to the mapping of muscle phenotypes with the objective to capture QTLs specific to muscle tissue. In the previous report, no correction for bone length was applied, hence leading to genetic architecture underlying more general growth, not necessarily specific to skeletal muscle (Lionikas et al. 2010). We explored how excluding the tibia length from the analysis would affect the genetic architecture presented in Table 3. This alteration in the LMM drastically reduced LOD scores across all muscles, and only one new QTL (affecting EDL, but not reported earlier (Lionikas et al. 2010)) was identified. This outcome was comparable to that of the analyses in the F34 intercross alone in which no QTL was identified (Lionikas et al. 2010). Hence controlling for variability in general growth is important for detection of QTLs which mainly influence skeletal muscle mass. Another possible cause for limited replication may reside in the combined use of the F2 and F34 intercrosses in the previous analysis (Lionikas et al. 2010). Alleles of closely linked relevant genes would tend to be inherited as a haplotype behaving as a single QTL in an F2 cross. Recombinations accumulated in an AIL would partition such QTL making detection of its constituent loci more difficult.

Four candidate genes were selected from replicated *Skmw34* locus (Chr 7); *Mical2, 2310014F06Rik, Arntl*, and *Sox6* emerged as promising candidate genes based on expression difference between the LG/J and SM/J strain muscle tissues (Lionikas et al. 2012), their known function and/or presence of nonsynonymous polymorphism in the coding sequence (Nikolskiy et al. 2015).

The *2310014F06Rik* is a bidirectional promoter long noncoding RNA (lncRNA) gene preferentially expressed in skeletal muscle. Several lncRNAs have been implicated in gene regulation during skeletal muscle differentiation (Li et al. 2012; Lu et al. 2013; Zhao et al. 2015). It has been also demonstrated that some lncRNA encode peptides with muscle‐specific expression which affect muscle contractility (Anderson et al. 2015; Nelson et al. 2016). The *2310014F06Rik* gene is differentially expressed between the two parental strains (Lionikas et al. 2010). As predicted by the strain comparison, the SM/J allele confers higher expression in comparison with the LG/J in the LG/SM AIL muscles (Fig. 4). Consistent with the putative regulatory role of lncRNAs, the *2310014F06Rik* gene was identified as a key driver of the LG/J and SM/J regulatory network (Lionikas et al. 2012). Examination of the RNA‐Seq data (Lionikas et al. 2012) revealed that ENSMUST00000211302.1, a splice variant of *2310014F06Rik* is expressed in the muscle. We then examined the genomic sequence of three regulatory elements, a promoter and two enhancers located in the proximity to this gene for polymorphisms between the LG/J and SM/J strains (Nikolskiy et al. 2015). Eleven indels were identified in total (Table 5) suggesting a putative cause for the difference in *2310014F06Rik* expression between the LG/J and SM/J alleles.

**Table 5:** Indel variants present in regulatory elements of the candidate genes

Gene	Strain	Position (bp)	Variant	Regulatory element	Identifier
*Dis3 l1*	SM	chr1:86821319	−C/−C	Promoter	ENSMUSR00000008214
	SM	chr1:86711651	+GTGTGTGT/+GTGTGT	Enhancer	ENSMUSR00000298578
*2310014F06Rik*	SM	chr7:112680305	+A/+A	Promoter	ENSMUSR00000249930
	SM	chr7:112681671	−A/−A	Promoter	ENSMUSR00000249930
	SM	chr7:112680104	+GCC/+GCC	Promoter	ENSMUSR00000249930
	SM	chr7:112687880	−CTTAC/−CTTAC	Enhancer	ENSMUSR00000249933
	SM	chr7:112687912	+GCGGGGA/+GCGGGGA	Enhancer	ENSMUSR00000249933
	SM	chr7:112687945	+C/+C	Enhancer	ENSMUSR00000249933
	SM	chr7:112687979	−ACTCGGAGGA/−ACTCGGAGGA	Enhancer	ENSMUSR00000249933
	SM	chr7:112688431	−TGTGTCGGC/−TGTGTCGGC	Enhancer	ENSMUSR00000249933
	SM	chr7:112688537	−C/−C	Enhancer	ENSMUSR00000249933
	SM	chr7:112689921	+TTGT/+TTGT	Enhancer	ENSMUSR00000249935
	SM	chr7:112690293	−A/−A	Enhancer	ENSMUSR00000249935
*Arntl*	SM	chr7:113207721	+CCGCCC/+CCGCCC	Promoter	ENSMUSR00000250055
	SM	chr7:113208434	−CTTCACTC/−CTTCACTC	Promoter	ENSMUSR00000250055
	SM	chr7:113206327	+T/+T	Promoter	ENSMUSR00000250055
	SM	chr7:113214962	−TA/−TA	Enhancer	ENSMUSR00000250056
	SM	chr7:113215089	−TTACTA/−TTACTA	Enhancer	ENSMUSR00000250056
*Smim15*	SM	chr13:108045634	−TTTTTTT/−TTTTTTT	Promoter	ENSMUSR00000080445

The LG/J strain sequence was use as the reference sequence.

The mRNA levels of the *Arntl* gene, a component of the circadian clock complex, were 1.7‐fold higher in TA muscle of the parental LG/J strain compared to the SM/J (Lionikas et al. 2012). The LG/J variant of the *Arntl* gene also conferred a higher expression of this gene compared to that of the SM/J in TA muscle of LG/SM AIL mice, suggesting that expression differences are driven by the local regulatory mechanisms. Strain comparison of the promoter and enhancer regions adjacent to *Arntl* (Nikolskiy et al. 2015) identified two indels in the promoter and two more in the enhancer which could be underlying expression difference (Table 5). Knockout of *Arntl* is characterized by smaller muscle fibers and increased proportion of oxidative fiber type in muscle tissue albeit no reported effect on muscle weight (Schroder et al. 2015).

*Sox6* also emerged as a positional candidate gene of particular interest because Quiat and colleagues reported that its knockout results in reduced myofiber size and decreased muscle weight (Quiat et al. 2011). Based on this knockout model, it is reasonable to hypothesize that reduction in *Sox6* expression would also lead to reduced muscle mass; however, the allele‐specific expression of *Sox6* (LG/J<SM/J, Fig. 4) and the allelic effect of *Skmw34* on muscle weight, LG/J > SM/J, were not consistent with such a scenario. Hence, *Sox6* is less likely to be the causative gene of *Skmw34*.

*Mical2* is highly expressed in the C2C12 cell line, but the expression of this gene was not significantly different between the LG/J and SM/J alleles. Additionally, there are no missense mutations, and therefore it is less likely that variation at *Mical2* explains the effect of the locus.

Two QTLs (Chr 1, 13), long bone 1.1 and 13.1 locus (lbn1.1b and lbn13.1), affecting tibia length overlapped with earlier findings in the same lineage (Norgard et al. 2011). Genes of interest reported by Norgard and colleagues (Norgard et al. 2011) included positional candidates from gene families known to contribute to skeletal growth. However, strain polymorphisms within these genes are unlikely to affect protein function (Nikolskiy et al. 2015). Candidate genes highlighted in this study (Table 4) are expressed in differentiated osteoclast (An et al. 2014), osteoblasts (Kansara et al. 2013) and/or chondrocytes (James et al. 2005, 2007; Fukada et al. 2008; Chau et al. 2014). Moreover, in silico analysis revealed deleterious polymorphisms within the coding sequence between the LG/J and SM/J.

Four candidate genes are expressed in chondrocytes and could affect tibia length (James et al. 2005, 2007; Fukada et al. 2008; Chau et al. 2014). The *Dis3l2* is located within the lbn1.1b; the SM/J allele of this gene possesses a missense mutation and 2 indels within a promoter and an enhancer elements of this gene (Table 5). Large mutations with breakpoints within *Dis3l2* have been found in humans with extreme skeletal phenotypes (Tassano et al. 2013), moreover an SNP (rs3103296) found within the gene was associated with human height (Lanktree et al. 2011). The *Dis3l2* was also found to be responsible for the Perlman syndrome and cancer susceptibility in humans (Astuti et al. 2012). The *Hjurp* gene is also located within the lbn1.1b locus, and a Pro508Arg polymorphism (LG/J allele shown first) is predicted to negatively affect protein function (Nikolskiy et al. 2015). The *Smim15* and *Srek1ip1* genes were prioritized in the Lbn13.1 locus (Nikolskiy et al. 2015). There is a damaging amino acid substitution (Trp61Arg) in *Smim15* and an indel in the promoter region (Table 5). The *Srek1ip1* function in the SM/J strain can be negatively affected by two damaging substitutions, Pro71Gly and Pro75Ser. These variations in the gene coding sequence could contribute to the phenotypic variation observed on the LG/SM AIL.

In conclusion, we replicated and identified novel QTLs affecting muscle weight and replicated bone length QTLs in the LG/SM AIL mice. Although the exact role of the underlying genes remains to be explored, our in silico and gene expression analyses in muscle tissue highlighted a manageable number of promising candidate genes for replicated QTL. As heritability estimates substantially exceed cumulative effect of the QTLs, a larger sample size will be required for further partitioning of missing heritability.

## Conflict of Interest

None declared.
